# Supplementary material for: Ending extreme poverty has a negligible impact on global greenhouse gas emissions
Source: Nature. 2023 Nov 29;623(7989):982–6. doi: 10.1038/s41586-023-06679-0 (PMC10686831; doi:10.1038/s41586-023-06679-0)
Supplement: Supplementary file 1 — Supplementary Discussion [file 41586_2023_6679_MOESM1_ESM.pdf]

nature portfolio

https://doi.org/10.1038/s41586-023-06679-0

Supplementary information

Ending extreme poverty has a negligible impact on global greenhouse gas emissions

In the format provided by the authors and unedited

Supplementary Information for “Ending extreme poverty has a negligible impact on global greenhouse gas emissions”

This file contains Supplementary Discussion. Part A.1 shows reductions in carbon in poor and non-poor countries needed to offset the emissions of poverty eradication. Part A.2 presents an extended scenario analysis and robustness checks.

A Supplementary Discussion

A.1 Offsetting the emissions of poverty alleviation in poor and non-poor countries

To calculate the decarbonization rate necessary to fully offset the additional greenhouse gases needed to end poverty, we first calculate the sum of additional greenhouse gases needed to end poverty until 2050:

$$ghgneeded_{world,total} = \sum_{y=2022,..,..,2050} \left(\sum_{c \in C_{poor}} ghgneeded_{c,y} \right)$$

Next, we calculate the greenhouse gas savings for poor or rich countries each year until 2050 if they become x% less carbon intensive annually (shown only for rich countries below):

$$ghgsavings_{rich}(x) = \sum_{y=2022,..,..,2050} \left[ghg_{rich,y} - ghg_{rich,y} * (1-x)^{y-2022} \right]$$

Finally, we set $ghgneeded_{world,total} = ghgsavings_{rich}(x)$ and solve for x. We do the same using poor countries instead of rich countries and at various poverty lines.

We can illustrate the relative efficacy of decarbonization in poor and non-poor countries using ISO-GHG curves (Extended Data Figure 6). The ISO-GHG curves show all combinations of reductions in carbon intensity in poor and non-poor countries that would offset entirely or in part the emissions from poverty alleviation. The intersection with the vertical axis is the reduction needed if only coming from non-poor countries alone, while the intersection with the horizontal axis is the reduction needed if only coming from poor countries.

To compensate for the additional emissions of ending extreme poverty in our baseline model, non-poor countries would need to decarbonize energy consumption at an average rate of 0.28% per year above and beyond the business-as-usual baseline scenario. In poor countries, an average additional decarbonization rate of 0.90% would be required to offset the additional emissions. In this scenario, any 1% reduction in carbon intensity in non-poor countries has the same effect on the emissions of poverty alleviation as a 3.2% reduction in carbon intensity in poor countries, reflecting the fact that most emissions accrue in non-poor countries.

At the lower-middle-income poverty line of $3.65 per day, decarbonization at a rate of 1% in non-poor countries and 2.15% in poor countries above the current trends would offset the additional emissions from poverty alleviation. Any 1% carbon intensity reduction in non-poor countries is equivalent a 2.2% reduction in poor countries.

To offset the emissions of poverty alleviation at the upper-middle-income poverty line of $6.85 per day, non-poor countries would need to decarbonize at a rate of 9.3% per year. This rate substantially exceeds decarbonization rates consistent with 1.5C or 2C warming trajectories, at 6.6% and 3.0% per year, respectively. In comparison, poor countries (relative to the $6.85 per

day poverty line) would need to decarbonize at a rate of 2.6% above business-as-usual. Any 1% emissions reductions in poor countries have the same effect as a 3.6% reduction in non-poor countries. When using the $6.85 poverty line, many more countries are considered poor than when using the extreme poverty line (Extended Data Figure 2a), and those countries emit most of the global emissions.

A.2 Scenario analysis

In the main text, we estimate emissions increases for scenarios in which inequality, energy intensity of GDP, and carbon intensity of energy consumption improve at best historical rates. Here, we expand the scenario analysis in several ways.

First, by looking at worst historical performers in energy efficiency, carbon intensity and inequality reduction. These are taken as the 90th percentile of the distribution of random effects for energy efficiency and carbon intensity, and as the 90th percentile of the changes to inequality observed (which is a 13% increase).

We further vary other key modelling parameters: the population growth forecasts until 2050 and passthrough rates from GDP growth to consumption growth. Baseline population projections are from the World Bank, whereas alternative scenarios are based on the low and high variant of the UN's Population Prospects (United Nation 2022). For the passthrough rates from GDP growth to consumption growth, we use the 10th and 90th percentile of the distribution of the random coefficients, which imply passthrough rates of 0.44 and 0.98.

We combine the best, worst, and default scenario of each of the five parameters (inequality, energy intensity, carbon intensity, population, passthrough rates), which gives us a total of 243 (=3^5) scenarios as are outlined in Extended Data Table 2. Note that for each of these scenarios, we do not only change the poverty alleviation projection but also the counterfactual projection. This means that when we, for example, switch from the baseline population projections to a low variant of population projections, we are looking at the greenhouse gas implications of ending poverty in a world where populations grow slower. We are *not* looking at the impact of ending poverty in a world with low population growth vis-à-vis a world with no growth and baseline population growth. Likewise, in the scenario with strong reductions in energy intensity, we are looking at the greenhouse gas implications of ending poverty in a world where countries reduce their energy intensity at the rate of historical best performers.

These different scenarios represent a wide range of outcomes with vastly different implications for emissions. For the extreme poverty line at $2.15, the emissions increase in 2050 relative to a scenario with no poverty alleviation ranges from 0.42% to 85%; for the $3.65 poverty line between 1.7% and 204%; and for the $6.85 poverty line between 6.1% and 611%. At all poverty lines, there are scenarios in which the emissions of poverty alleviation are modest (Extended Data Figure 8a). At the extreme poverty line, 114 scenarios have estimated emissions increase in 2050 of less than 5%, but there are also 26 scenarios below 5% at the $3.65 poverty line, and even at the $6.85 poverty line, there are 8 scenarios with emissions increases between 5% and 10%. These optimistic scenarios, however, are contingent on combining low inequality and green growth pathways (Extended Data Figure 8b).

On the flipside, there are scenarios with very significant emissions increases even at the $2.15 line, which are combinations of rising inequality, increasing energy intensity of GDP, and increasing carbon intensity of energy. The average emissions of all extreme poverty scenarios

with rising inequality are 18.7%, 15.4% with increasing energy intensity of GDP, and 17.0% with increasing carbon intensity of energy (Extended Data Figure 8b).

We also run another set of scenarios to get a sense of some of the uncertainty implicit in our modeling approach. Concretely, for the three equations where we rely on a mixed model, we use the point estimates of the fixed and random effects along with their standard errors to create distributions of fixed effects and (country-specific) distributions of random effects. We draw from these distributions 1000 times, for each draw estimate the emissions associated with poverty alleviation, and create a histogram of these results at the three poverty lines. The point estimate and confidence intervals at the three lines are 4.9% [4.1%-10.8%], 15.3% [13.0%-38.3%], 45.7% [40.4%-129.6%] (Extended Data Figure 8c). The results are qualitatively unchanged --- ending extreme poverty does not require a massive addition to global emissions, while ending poverty at higher lines does with historical rates of improvements in carbon and energy efficiency, especially at the upper middle-income line.

It is worth noting that the confidence bands mentioned above do not account for all uncertainty. There is also uncertainty related to the projection of the welfare distributions, the imputations of data for countries without primary data, the population numbers, etc. The uncertainty reported above would decrease if we winsorized the random coefficients more.

In general, if we do not winsorize the random effects in any of the regressions and just use the raw random effects, our baseline additional emissions in 2050 relative to 2019 levels for the three poverty lines increase from 4.9% to 6.5% ($2.15), from 15.3% to 18.7% ($3.65), and from 45.7% to 52.9% ($6.85). If we winsorize at the 25th and 75th percentile instead, our results (mostly) decrease to 5.0%, 14.9%, 41.9%.

Finally, we construct one other scenario where we model population growth rather than take it from the UN. We do so because the countries that are not projected to grow sufficiently to end poverty by 2050, and for which we model growth to increase such that the poverty target is precisely met by 2050, this added economic growth would likely imply that fertility would fall faster than in our baseline projections. For the countries that are likely to end poverty before 2050 with current growth forecasts, we do not need to add any additional growth as the baseline population scenarios are likely already accounting for the impact of expected economic development on fertility.

To account for the impact of economic growth on fertility, we first run an exponential regression of population growth on log GDP per capita, which returns the following fit: popgrowth = 201.08*0.25^log10(GDP/capita) and an R^2 of 0.67. Next, we predict a country's population growth using the GDP/capita level needed to end poverty by 2050 and using the GDP/capita expected with current growth forecasts. For the countries where the GDP/capita needed to end poverty is higher than current growth forecasts, we take the difference in the two predicted population growth rates and use it to adjust downwards the baseline population growth. Doing so pushes down the emissions of poverty alleviation relative to our main results (Extended Data Figure 9c), but not enough to change our conclusions.
